# Fast effectiveness of a solubilized low‐dose budesonide nasal spray in allergic rhinitis

**DOI:** 10.1111/cea.13691

**Published:** 2020-07-07

**Authors:** Petra Zieglmayer, René Schmutz, Patrick Lemell, Nicole Unger‐Manhart, Sabine Nakowitsch, Andreas Goessl, Markus Savli, René Zieglmayer, Eva Prieschl‐Grassauer

**Affiliations:** ^1^ Power Project GmbH Vienna Challenge Chamber Vienna Austria; ^2^ Medical University Vienna Austria; ^3^ Medical School Sigmund Freud University Vienna Austria; ^4^ Marinomed Biotech AG Vienna Austria; ^5^ Biostatistik & Consulting Hartberg Austria

**Keywords:** allergic rhinitis, budesonide, challenge chamber, early onset, inflammation, intranasal glucocorticoids, nasal spray, preservative‐free

## Abstract

**Background:**

Budesonide, a poorly water‐soluble corticosteroid, is currently marketed as a suspension. Budesolv is a novel aqueous formulation containing dissolved budesonide showing increased local availability in preclinical models. Budesolv contains ~85% less corticosteroid than the marketed comparator.

**Objective:**

The study (EudraCT:2018‐001324‐19) was designed to assess non‐inferiority of Budesolv compared to Rhinocort® Aqua 64 (RA) and early onset of action.

**Methods:**

In a three‐way cross‐over double‐blinded randomized trial, Budesolv 10 was compared to RA and placebo in grass pollen allergic rhinoconjunctivitis volunteers (n = 83 (ITT); n = 75 (PP)). On day 1, participants entered the Vienna Challenge Chamber (VCC) for 6 hours; first treatment took place at 1:45 hours after entry. Participants treated themselves for further 6 days; on day 8, the last treatment was applied before entering the VCC. Subjective symptom scores, nasal airflow and nasal secretion were measured regularly during allergen challenge.

**Results:**

Budesolv 10 was equally effective compared to RA with respect to TNSS and nasal airflow after eight days of treatment with a strongly reduced dose (more than 80% reduction). After first dose, only Budesolv 10 showed a significant reduction of nasal and respiratory symptoms starting 90 minutes (*P* < .05) and 15 minutes (*P* < .05) after application onwards, respectively, demonstrating an early onset of efficacy. A clinically significant 1 point reduction in nasal symptom score was reached at 195 minutes (*P* < .05) after application.

**Conclusions and clinical relevance:**

The novel preservative‐free, aqueous low‐dose budesonide formulation is highly efficacious even after an initial single treatment. Thus, Budesolv 10 appears to be an effective acute treatment for allergic rhinitis as well as for AR comorbidities like mild asthma and conjunctivitis.

**Abbreviations**
AE: adverse event
AR: allergic rhinitis
CI: confidence interval
ECC: environmental challenge chamber
ECG: electrocardiogram
FEV1: forced expiratory volume in 1 second
ITT: intent‐to‐treat population
LPS: lipopolysaccharide
PP: per‐protocol population
RA: Rhinocort® Aqua 64 nasal spray
RTSS: Rhinitis total symptom score
TNF‐alpha: tumour necrosis factor‐alpha
TNSS: Total Nasal Symptom Score
TOSS: Total Ocular Symptom Score
TRSS: Total Respiratory Symptom Score
VCC: Vienna Challenge Chamber

## 1 INTRODUCTION

Allergic rhinitis (AR), either perennial or seasonal, is one manifestation of a type I hypersensitivity reaction caused by an immune reaction to otherwise innocuous agents such as pollen or house dust mites. In the past decades, a globally rising trend of AR has been observed with widely varying prevalence particularly in the developing countries. Up to one‐quarter of the global population may be affected.^1^ While allergic rhinitis refers to an inflammatory process of the nasal passages, symptoms involve the nose and may extend beyond to affect the eyes, ears, sinuses and bronchi. Commonly reported nasal symptoms include nasal itching and congestion, runny nose and sneezing. Often, AR will involve the conjunctivae; such patients may experience itching, tearing and red eyes. About 40% of AR patients also suffer from asthmatic symptoms like cough, wheeze and dyspnoea.^2^ In fact, AR is considered an independent risk factor for subsequent asthma.^3^

Apart from allergen avoidance and physical measures, current therapy of AR comprises two main treatment options: allergen immunotherapy or pharmaceuticals targeting the consequences of mast cell mediator release. While immunotherapy is the only treatment with a long‐term sustained effect that is intended to result in a reduced reactivity to the respective allergen, pharmaceutical interventions either block histamine from binding to its receptor (anti‐histamines), stabilize mast cells (eg cromoglycate), or reduce the release of pro‐inflammatory mediators such as TNF‐alpha. The latter is achieved with the topical application of corticosteroids such as budesonide, fluticasone propionate or others. Corticosteroids are synthetic analogues of cortisol and bind intracellular receptors resulting in the anti‐inflammatory action. After binding to the drug, the receptor molecules are transferred into the nucleus of the cells and bind to promoter regions of target genes. In case of pro‐inflammatory mediators, binding of a glucocorticoid receptor to the promoter induces a reduction of gene transcription into mRNA and thereby a reduction of synthesis and release of such mediators. Corticosteroids are potent substances, but their intranasal application is hampered by the fact that new generation corticosteroids are highly hydrophobic and therefore generally applied as suspensions. Budesonide is a steroid widely used in the indications of AR and allergic asthma. It is well established that the use of steroids is beneficial for patients suffering from AR, because adequate treatment provides disease control preventing the worsening of the underlying disease. While first‐generation steroids, such as dexamethasone phosphate, are soluble in aqueous media including plasma and therefore exert systemic side effects, newer drugs including budesonide, fluticasone propionate and others are hardly soluble in water and therefore detected only in minimal amounts systemically.^4^ In addition, budesonide is inactivated by the liver during the first passage by degradation via the cytochrome pathway. The low solubility, and the application of the drugs as suspensions as a consequence thereof, results in a lag time of up to several days until efficacy can be observed, a situation that is unsatisfactory for patients suffering from allergic symptoms. The poor bioavailability of suspended drugs is also illustrated by a study where different doses of budesonide in suspension (64 µg vs 256 µg per day) have been applied in patients suffering from AR: both doses were similarly effective suggesting that increased amounts of undissolved drug do not contribute efficiently to the clinical response.^5^

Different combinations of corticosteroids and anti‐histamines have been evaluated in clinical trials, showing superior efficacy compared to either of the components alone,^6^, ^7^, ^8^ but for only one of these combinations a market authorization and real‐world evidence exist yet.^9^, ^10^ The lag time is shortened, as the anti‐histamines exert their function early on, while the corticosteroids address the inflammation later. The corticosteroid in these combinations is still presented as suspension, and thereby, most of the applied drug is not active in the nasal cavity but swallowed and transferred into the system.^4^

Another pharmaceutical development strategy is increasing the solubility of corticosteroids. Actually, Captisol, a β‐cyclodextrin, was used to solubilize budesonide in a nasal formulation. In two environmental challenge chamber studies, therapeutic equivalence of both nasal sprays, solubilized budesonide (640 µg/mL) nasal spray and marketed suspended nasal spray Rhinocort® aqua 64 was tested; in a pooled analysis, equivalence of dissolved and suspended budesonide in the same concentration was demonstrated.^11^

Recently, a novel proprietary combination of known excipients was developed that allows the solubilization of budesonide.^12^ The new budesonide formulation has been tested preclinically in vitro, ex vivo and in vivo with respect to permeation into cells and tissue, as well as effectivity in a lung inflammation model in comparison with the marketed product Rhinocort® Aqua 64 nasal spray (RA). Although the concentration of budesonide in this new formulation is much lower than in RA, a significant decrease of inflammatory mediator release compared to placebo or marketed product could be shown in an acute lung inflammation model, if applied 24 hours, 18 hours or 3 hours before an LPS challenge [Nakowitsch et al, manuscript in preparation]. More importantly, the solubilized budesonide formulation also significantly reduced TNF‐alpha in bronchoalveolar lavage, if applied 15 minutes after the challenge compared to placebo and marketed product, reflecting the fast availability of the drug in the target tissue.^12^

The aim of the current study was to verify the promising preclinical results shown for Budesolv in a pivotal clinical trial. We hypothesize rapid onset of action of Budesolv with regard to individual symptom scores in patients with AR as well as equal efficacy in prolonged treatment compared to marketed product.

## 2 MATERIALS AND METHODS

This study (EudraCT:2018‐001324‐19) was conducted at the Vienna Challenge Chamber in compliance with International Conference on Harmonization guidelines and in accordance with the Declaration of Helsinki. The protocol received clearance from the Austrian Competent Authority as well as the ethics committee of the Sigmund Freud University Vienna. Written informed consent was obtained from all patients.

### 2.1 Study patients

Study participants were above 18 years of age, with a documented clinically relevant history of moderate to severe seasonal allergic rhinitis to grass pollen for the previous 2 years. Briefly, all volunteers demonstrated a positive skin prick test response to grass pollen and a positive serum IgE result at screening or during the previous 6 months. Furthermore, they had a positive subjective response to a grass pollen challenge during a screening run. Detailed information on inclusion and exclusion criteria is given in the supplemental materials.

### 2.2 Study medication

In the marketed comparator Rhinocort® Aqua 64 nasal spray, budesonide is presented as suspension with a concentration of budesonide of 1280 µg/mL. Budesolv 10 contained 200 µg/mL budesonide solubilized in an aqueous formulation. Placebo is based on the composition of Budesolv 10 without active ingredient or the excipients required for the solubilization thereof.

Budesolv 10 and placebo were filled in a primary container system using a nasal spray pump identical to the one used for Rhinocort Aqua 64. For blinding purposes, all primary packages were visually identical, and additionally wrapped entirely in an opaque self‐adhesive label to prevent unblinding on the basis of the appearance of the product itself (Budesolv 10 and placebo are clear liquids, whereas Rhinocort Aqua is a milky‐white suspension).

### 2.3 Study design

**Figure 1:**
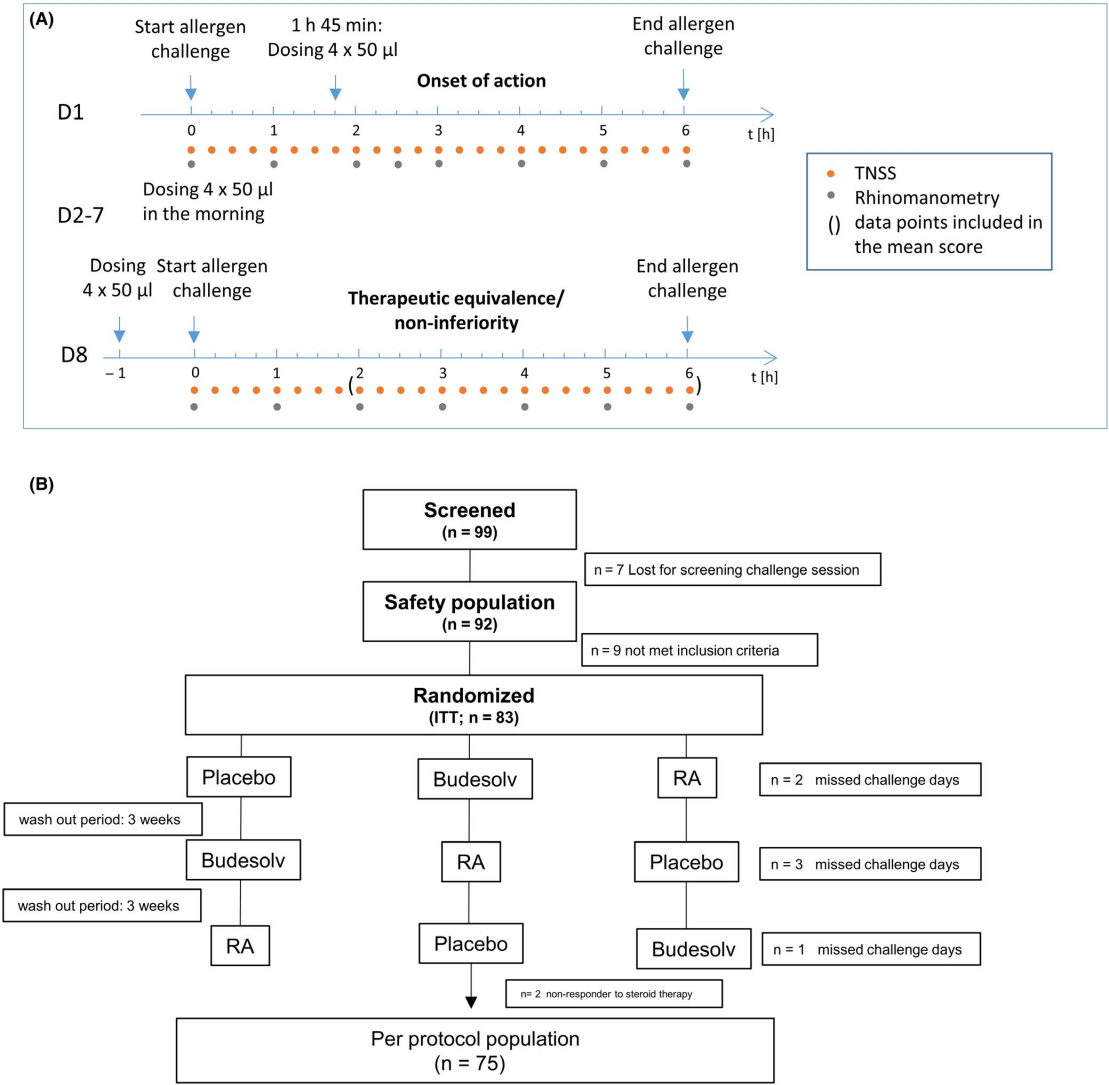
A, Study outline of the phase III clinical study on days 1 and 8 comparing Budesolv with Rhinocort® Aqua 64 and placebo. On day 1, volunteers were exposed to grass pollen in an environmental challenge chamber for six hours. After 1.45‐2 hours, participants received the first treatment (2 puffs per nostril, ie 200 µL total) resulting in a residual observation period of 4.15 hours. On day 8, after eight days of treatment, participants were exposed to grass pollen for six hours. 1 hour prior exposure, the last dosing of the nasal spray was applied. At both days, subjective symptoms were recorded every 15 minutes (orange dots), rhinomanometry (grey dots), and nasal secretion was evaluated every hour or 30 minutes, respectively. B, Study populations. Ninety‐nine subjects were screened resulting in 92 volunteers being eligible for the safety population. Eighty‐three volunteers started the active phase of the study and finished at least one cycle of the trial comprising the intent‐to‐treat population (ITT). Seventy‐five participants were eligible for the per‐protocol population (PP)

This was a randomized, double‐blind, placebo‐controlled three‐way cross‐over study to evaluate a non‐inferior therapeutic effect of Budesolv 10 compared to RA after 8 days of treatment. A washout interval of 3 weeks was kept between treatment sequences. According to the patient information leaflet of RA, a dose of 256 µg/d is needed to reach maximal therapeutic effect after 7‐14 days of treatment. Therefore, to demonstrate non‐inferiority to RA, participants were treated for 8 days. Furthermore, an early onset of action was evaluated after application of the first dose. The study was conducted between October 2018 and April 2019. One to four weeks prior to first treatment period, each volunteer underwent a 6‐hour screening grass pollen challenge session in the Vienna Challenge Chamber (VCC) to ensure a TNSS of at least 6 points out of 12 within the first two hours and a persistent response to the challenge. On day 1 of the first treatment period, eligible volunteers were randomized to one of the three treatment sequences (Placebo: Budesolv 10: RA = 1:1:1). For determination of onset of action, participants entered the VCC for 6 hours and received their first treatment 1 hour and 45 minutes after challenge start to assess the onset and the magnitude of symptom relief after first dose. The treatments were given as two actuations of 50 µL each per nostril and continued at home for further 6 days in the morning. On day 8 of each treatment period, participants applied their last treatment one hour prior to a 6‐hour grass pollen allergy challenge (Figure 1A).

### 2.4 Allergen challenge

A 6‐hour grass pollen allergen challenge was carried out at screening, on days 1 and 8 of each treatment period in the Vienna Challenge Chamber (VCC), using a validated method.^5^, ^13^ The VCC can accommodate up to 20 subjects in one session, all of whom were under constant supervision by, and could communicate with, medical staff outside the chamber.

Communication was possible through clear glass windows and via an intercom system. During the challenge, the chamber is charged with 100% fresh air, which is conditioned (filtered, heated, dried, cooled and humidified) and then loaded with a qualitatively and quantitatively determined pollen amount. The challenge agent used in the chamber was a mixture of four grass pollen species (Timothy, Orchard, Perennial rye and Sweet vernal grass) (Allergon SB, Sweden). Air temperature (24°C), humidity (40%) and allergen load (approximately 1500 grains per cubic meter) were constantly monitored and maintained.

During the seven grass pollen challenge sessions, participants scored their subjective symptom scores—Total Nasal Symptom Score (TNSS); Total Ocular Symptom Score (TOSS); Total Respiratory Symptom Score (TRSS)—every 15 minutes. As objective parameter, nasal airflow and nasal secretion weight were assessed every 60 minutes and every 30 minutes, respectively. On day 1, an additional nasal airflow measurement was performed about 45 minutes after treatment (see Figure 1).

### 2.5 Evaluation of subjective symptom scores

#### 2.5.1 Total nasal symptom score (TNSS)

TNSS is the sum of the single symptom scores of nasal congestion, rhinorrhoea, nasal itching and sneezing. Each single symptom was scored on a four‐point categorical scale from 0 to 3 (where 0 = absent symptoms, 1 = mild symptoms, 2 = moderate symptoms, and 3 = severe symptoms) giving a TNSS from 0 to 12.

#### 2.5.2 Total ocular symptom score (TOSS)

TOSS is the sum of the single symptom scores of conjunctival redness, itchiness and tearing. Each single symptom was scored on a four‐point categorical scale from 0 to 3 (where 0 = absent symptoms, 1 = mild symptoms, 2 = moderate symptoms, and 3 = severe symptoms), giving a total ocular symptom score (TOSS) from 0 to 9. The score represented an average for both eyes.

#### 2.5.3 Total respiratory score (TRSS)

TRSS is the sum of the single symptom scores of cough, wheeze and dyspnoea. Each single symptom was scored on a four‐point categorical scale from 0 to 3 (where 0 = absent symptoms, 1 = mild symptoms, 2 = moderate symptoms, and 3 = severe symptoms), giving a total respiratory symptom score (TRSS) from 0 to 9.

### 2.6 Evaluation of nasal airflow with active anterior rhinomanometry

Nasal airflow was determined by using active anterior rhinomanometry at a pressure difference of 150 Pascal across the nasal passages (sum of the right and left nostril values) immediately before (baseline) and every 60 minutes during allergen exposure in the VCC, both on day 1 (baseline) and on day 8 of each study period (6 hours). On day 1 of each treatment period, 45 minutes after the first dose of medication a further assessment was done.

### 2.7 Evaluation of nasal secretion weight

Pre‐weight paper tissues were collected every 30 minutes, and nasal secretion weight was determined in grams.

### 2.8 Safety

Adverse events (AEs) were monitored throughout the entire duration of the study starting from screening after signature of informed consent. Vital signs (heart rate and blood pressure) were measured at the screening visit, and on each allergen challenge day pre‐dose and immediately after leaving the challenge chamber. Routine haematology, blood chemistry and urinalysis assessments, and single 12‐lead ECGs were assessed at the screening and follow‐up visit. FEV1 was measured on all allergen challenge study days pre‐challenge, every 2 hours during the session and at the end of the allergen challenge.

### 2.9 Statistical analysis

A sample size of at least 72 subjects was estimated based on the non‐inferiority margin of 15 percent points for the TNSS assuming a standard deviation of 35%‐40% and a power of at least 80%. Considering a dropout rate of 10%‐15%, up to 100 subjects were planned to be screened to randomize about 84 volunteers to get at least 72 evaluable participants for analysis.

The primary study end‐point was the mean “Total Nasal Symptom Score” (TNSS), calculated as mean of TNSS measured at 17 time points (every 15 minutes) during the grass pollen allergen exposure challenge in the time window of 2 to 6 hours on day 8. The hypothesis to be tested was non‐inferiority of Budesolv 10 in comparison with RA. The null hypothesis is defined as

$$\text{Mean TNSS}\;2‐6h\left(\text{Test}\right)>\left[\text{Mean TNSS}\;2‐6h\left(\text{reference}\right)+15\%\right]$$

The alternative hypothesis is defined formally as

$$\text{Mean TNSS}\;2‐6h\left(\text{Test}\right)\leq \left[\text{Mean TNSS}\;2‐6h\left(\text{reference}\right)+15\%\right]$$

Non‐inferiority was concluded if the upper limit of the 95% confidence interval of the mean TNSS of Budesolv does not exceed 15% of the upper limit of the 95% confidence interval of the mean TNSS difference of Budesolv compared to the active comparator originating from the mean TNSS of the active comparator (ie the non‐inferiority margin).

As secondary study end‐point, the onset of action was calculated as mean difference of TNSS to pre‐treatment baseline defined as the mean of the three last observations before treatment (ie at 1:15, 1:30 and 1:45 hours after chamber session start) during the first allergen challenge (day 1). Onset of action was assumed when the first time point of the mean TNSS difference between the active treatment and placebo is *P* < .05. Additional secondary efficacy end‐points included mean nasal air flow, mean nasal secretion, mean TOSS and mean TRSS, respectively.

Intention‐to‐treat (ITT) and per‐protocol (PP) populations were predefined. The per‐protocol population will assess the effect of treatment on patients who are compliant with the protocol which comprises all patients with data obtained without major protocol deviations and will be the primary population of interest for the non‐inferiority analyses.

Testing was carried out with type I error controlled at a two‐sided alpha level of 0.05 and point estimates with 95% CIs. There was no adjustment for multiplicity testing of the variables. Statistical analysis was performed with R 3.4.^14^

## 3 RESULTS

### 3.1 Patient population

A total of 99 grass pollen allergic patients underwent a screening procedure with 92 patients included in a six‐hour grass pollen challenge (safety population). A total of 83 patients reached minimum scores of six points within 2 hours and were thereby eligible for the study. Five volunteers did not complete the cycles on day 1, and one additional participant did not complete the cycles on day 8, resulting in an evaluable ITT population of 78 (day 1) and 77 (day 8), respectively. Furthermore, two participants did not show any TNSS reduction in any cycle on day 8 and were therefore regarded as previously not discovered non‐responders to steroid treatment and thus excluded from the PP population which then comprised 75 subjects for both, day 1 and day 8 (Figure 1B). Exclusion of the non‐responding participants was decided before unblinding and was in accordance with the predefined exclusion criteria. Results were calculated for both study populations (ITT and PP), but data presented as figures for early onset of action (day 1) are presented for the ITT population, whereas data presented as figures for non‐inferiority (day 8) are shown for the PP population (PP; Figure 1B). This approach is in accordance with FDA Guidance for Industry for non‐inferiority clinical trials to establish effectiveness.^15^

Demographic data of all randomized volunteers (N = 83) (Table 1) show that 60% of the participants were female and 40% were male. The median age was 31 years, and the median body mass index was 22.84 kg/m^2^.

**Table 1:** Demographic and other baseline characteristics

Characteristic	female	male	total
Age			
Mean	32.56	34.06	33.16
Median	31	30	31
Range	20‐61	20‐59	20‐61
Gender	50 (60.24%)	33 (39.76%)	83(100%)
Height (cm)			
Mean	166.76	180.00	172.02
Median	166	180	172
Range	154‐180	170 ‐ 190	154‐190
Weight (kg)			
Mean	63.98	77.39	69.31
Median	62.5	78	68
Range	50‐90	58‐100	50‐100
BMI (kg/m^2^)			
Mean	22.98	23.88	23.34
Median	22.18	23.55	22.84
Range	19.20‐29.76	19.61‐29.75	19.20‐29.76
Duration of SAR (yrs)			
Mean	20.90	23.36	21.88
Median	19	22	21
Range	6‐42	8‐41	6‐42
SPT (diameter, mm)			
Mean	9.10	10.00	9.46
Median	9.0	9.0	9.0
Range	5.0‐15.0	5.0‐21.0	5.0‐21.0

### 3.2 Budesolv is equally effective compared to the marketed comparator with respect to TNSS after eight days of treatment

**Figure 2:**
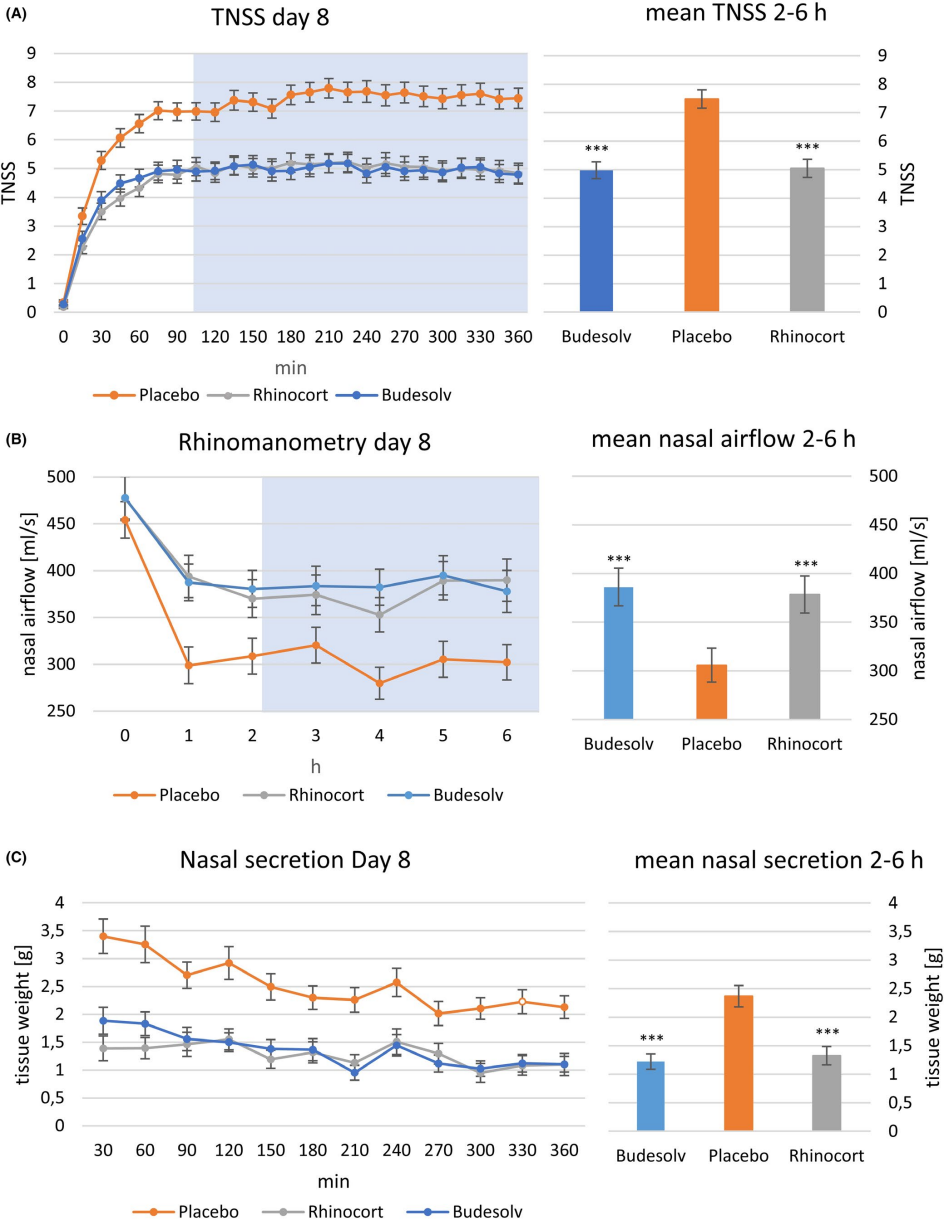
TNSS (A), Rhinomanometry (B), Nasal secretion (C) after eight days of treatment with either Budesolv, Rhinocort® Aqua 64, or a placebo nasal spray over a time period of 6 hours (left panel, x‐axis). Mean values between 2 and 6 hours are shown in the right panel. Each data point represents the mean of the values from participants eligible for the PP population. The blue‐shaded area shows the time period applicable for the evaluation of the primary end‐point (2‐6 hours). *** means significant difference to placebo with *P* < .001

In Figure 2A, the time course of mean TNSS over six hours for each treatment is shown (left panel, PP population). Both active treatments significantly reduced the mean TNSS compared to placebo (right panel, *P* < .001). Equivalence of TNSS was demonstrated in both populations, PP and ITT: mean difference of TNSS (RA–Budesolv) was 0.07 ± 2.48 (PP) and 0.08 ± 2.53 (ITT) respectively; the upper limit of the 95% confidence interval of the difference was 0.64 and 0.63; the mean TNSS of Budesolv (4.98 (PP); 5.08 (ITT)) plus the confidence interval of the differences (0.64 and 0.63) was below the mean of mean TNSS of RA plus 15% predefined non‐inferiority margin (5.80 (PP); 5.93 (ITT)).

### 3.3 Nasal airflow and nasal secretion support the effectiveness of Budesolv

**Figure 3 1:**
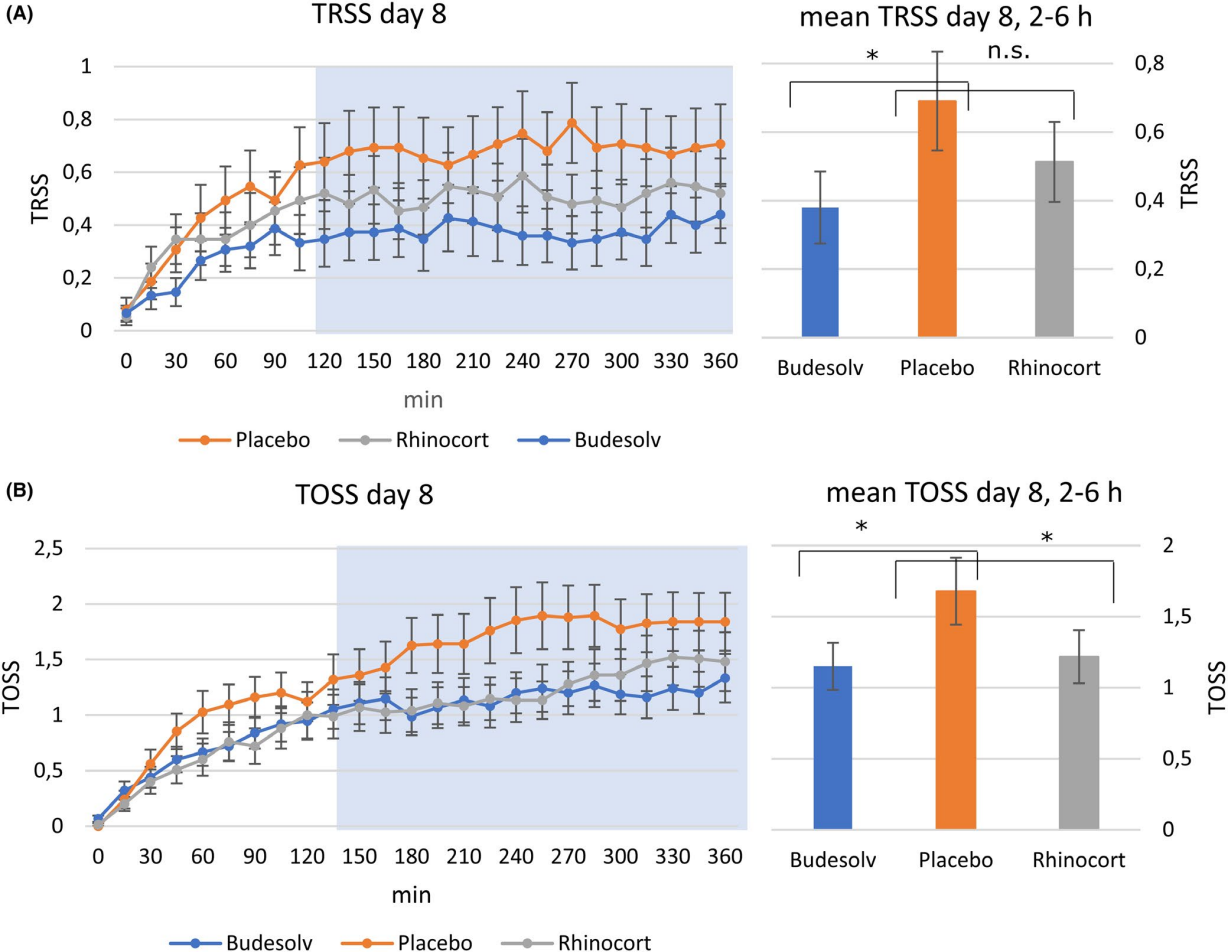
Mean TRSS (A) and mean TOSS (B) after eight days of treatment with either Budesolv, Rhinocort® Aqua 64, or placebo nasal spray over a time period of 6 hours (left panel, x‐axis). Mean values between 2 and 6 hours are shown in the right panel. Each data point represents the mean of the values from participants eligible for the PP population. The blue‐shaded area shows the time period applicable for the evaluation of the primary end‐point (2‐6 hours). * means *P* < .05, n.s. means not significant

As an objective parameter to demonstrate non‐inferiority between Budesolv and RA as well as superiority compared to placebo, anterior nasal airflow measured by rhinomanometry was used. The time course for mean values of nasal airflow of each treatment is shown in Figure 2B, left panel for PP population. Both active treatments showed significantly improved values in nasal airflow (*P* < .001) in both populations, PP (Figure 3B, right panel) and ITT. Similar to the results with TNSS, also for patients treated with Budesolv non‐inferiority to RA was shown regarding nasal airflow in both populations.

In addition to nasal airflow measurement, the determination of nasal secretion weight served as a second objective parameter. Supporting the results of the TNSS and nasal airflow, nasal secretion weight also was significantly reduced after eight days of treatment with either Budesolv or RA compared to placebo in the PP (Figure 2C; right panel, *P* < .001) and ITT population. Data showing the time course of nasal secretion weight reflecting the PP population are shown in Figure 2C, left panel. Although the mean values for nasal secretion weight after RA and Budesolv treatment showed a high level of concordance, non‐inferiority could not be demonstrated due to the immanent large variability of the data (coefficient of variation = 96%).

### 3.4 Budesolv is superior compared to the marketed comparator and significantly reduces respiratory symptoms (TRSS)

In Figure 3A, left panel, the time course of the means of TRSS is shown for each study medication. The mean value between 2 hours and 6 hours determined for Budesolv was significantly lower compared to the mean value of placebo (*P* = .02 for both PP and ITT, Figure 3 A, right panel) while a non‐significant improvement was observed for RA (*P* = .19 for both PP and ITT), (Figure 3A, right panel). The mean TRSS difference between Budesolv and RA did not reach significance (*P* = .24).

### 3.5 Both active treatments significantly reduce ocular symptoms (TOSS)

As for TRSS, the time course of the mean values of TOSS is shown in Figure 3B, left panel. Both active treatments showed a significant reduction of mean TOSS between 2 hours and 6 hours (*P* = .01 and *P* = .04 (PP) and *P* = .02 and 0.04 (ITT), for Budesolv and RA, respectively; compare Figure 3B, right panel). There was no significant difference in mean TOSS between Budesolv and RA (*P* > .05).

### 3.6 Budesolv enables a fast onset of action after first dose

**Figure 4:**
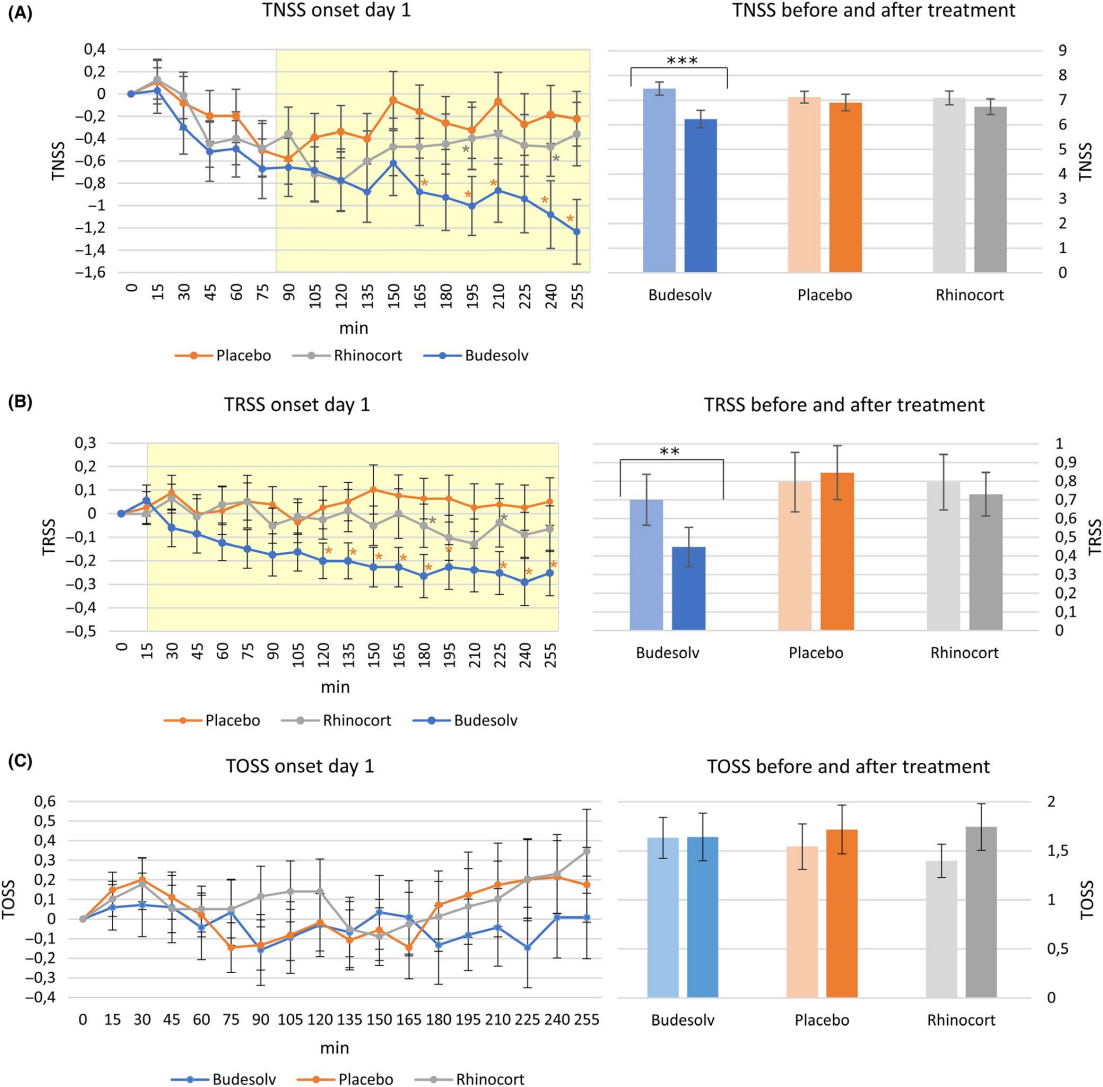
Right panels: TNSS (A), TRSS (B), or TOSS (C) before and after treatment. The light‐coloured bars represent mean values of the last three time points before treatment, and the dark bars represent the mean after 4.15 hours treatment. *** means *P* < .001, ** means *P* < .01. All values represent means of all volunteers eligible for the ITT population. Left panels: onset of action of Budesolv compared to Rhinocort® Aqua 64 or placebo with respect to TNSS (A), TRSS (B) or TOSS (C; y‐axis). Duration of challenge after treatment is indicated at the x‐axis. Onset of action was calculated using the mean of the three last time points before treatment as baseline. Values with * indicate time points with a significant difference between Budesolv and placebo (*), or Budesolv and Rhinocort® Aqua 64 (*); the shaded area indicates the observation period where the mean TNSS reduction of participants treated with Budesolv is significantly stronger compared to the TNSS reduction of participants treated with placebo. All values represent means of all volunteers eligible for the ITT population

Figure 4A left panel shows the time course of TNSS after the respective treatments referenced to the pre‐treatment baseline values. Ninety minutes after the first application, the effectiveness until the end of the observation period of Budesolv in comparison with placebo becomes significantly different (mean score between 90 minutes and 255 minutes, yellow shaded). Comparing Budesolv and placebo at single time points showed a significant reduction of TNSS for the first time after 165 minutes (2:45 hours), *P* = .047. RA treatment did not show significant reductions of TNSS during the observation period, neither with respect to summarized means nor at single time points compared to placebo (*P* > .05). The rapid efficacy onset of Budesolv is even further underlined by significant reductions of mean TNSS compared to RA at time points 195 minutes (*P* = .04) and 255 minutes (*P* = .006) after first application. At the last time point of continuous observation (4.15 hours after first dose), volunteers treated with Budesolv reported a total reduction of symptom scores of approximately −1.2 ± 2.6 points compared to pre‐treatment baseline, while both placebo and RA only marginally reduced the symptom scores (−0.2 ± 2.2 and −0.4 ± 2.5 points, respectively; Figure 4A, right panel). Comparing the TNSS before and after treatment, Budesolv showed a significant mean reduction of 1.24 symptom points (95% CI; −1.81 ‐ −0.66; *P* < .001 for both ITT and PP), while RA and placebo showed no significant reduction (*P* = .21 and *P* = .37, respectively; Figure 4A, right panel).

Figure 4B left panel shows the time course of TRSS after the respective treatments referenced to the pre‐treatment baseline values. Considering the total observation period, Budesolv compared to placebo induced an immediate significant TRSS decrease starting from the first time point after application (*P* = .028 ITT and PP, yellow shaded). Single time point analysis showed successive significant reductions of TRSS beginning at 120 minutes after Budesolv treatment in comparison with placebo (all *P* < .05) except for time point 210 minutes (*P* = .056). In contrast, RA treatment did not reveal any significant TRSS reduction. Moreover, mean Budesolv TRSS reduction vs RA was significantly higher pronounced at time points 180 minutes (*P* = .047) and 225 minutes (*P* = .042).

At the last point of continuous observation (4.15 hours after first dose), participants treated with Budesolv reported a significant reduction of TRSS of approximately 0.25 points (95% CI; −0.45‐−0.06; *P* = .01 for ITT and PP) compared to pre‐treatment baseline, while both placebo and RA showed no significant reduction (*P* = .51 and *P* = .61, respectively; Figure 4B, right panel). In contrast to TNSS and TRSS, TOSS was not statistical significantly affected by any treatment on day 1 (*P* > .05; Figure 4C, left and right panels).

### 3.7 Safety

The safety population consisted of 92 subjects. The assessment of safety does not show any significant differences between the three treatment groups with respect to severity of AEs, drug relationship or action required (*P* > .05, compare Table 2). In the Budesolv 10 group, 3 possible related and 1 unlikely related AE occurred whereas in the RA group 1 possible related and 3 unlikely related AEs were found. In the placebo group, 1 possible related and 3 probable related AEs were seen (Table S1).

**Table 2:** Assessment of safety

	Screening	Budesolv 10	Rhinocort ® Aqua 64	Placebo	Overall	Fisher Test
	N (%)	N (%)	N (%)	N (%)	N (%)	
Adverse Events	1 (100%)	22 (100%)	19 (100%)	19 (100%)	61 (100%)	
Severity						
Mild	0 (0%)	4 (18.2%)	5 (26.3%)	7 (36.8%)	16 (26.2%)	*P* = .84
Moderate	1 (100%)	17 (77.3%)	13 (68.4%)	11 (57.9%)	42 (68.9%)	
Severe	0 (0%)	1 (4.5%)	1 (5.3%)	1 (5.3%)	3 (4.9%)	
Drug Relationship						
Not related	1 (100%)	18 (81.8%)	15 (78.9%)	15 (78.9%)	49 (80.3%)	*P* = .24
Possible*	0 (0%)	3 (13.6%)	1 (5.3%)	1 (5.3%)	5 (8.2%)	
Probable^#^	0 (0%)	0 (0%)	0 (0%)	3 (15.8%)	3 (4.9%)	
Unlikely	0 (0%)	1 (4.5%)	3 (15.8%)	0 (0%)	4 (6.6%)	
Action						*P* = .16
None	0 (0%)	4 (18.2%)	4 (21.1%)	9 (47.4%)	17 (27.9%)	
Treatment given	1 (100%)	18 (81.8%)	15 (78.9%)	10 (52.6%)	44 (72.1%)	

Note

The number of AEs during screening and in the different treatment groups has been assessed with respect to severity, drug relationship and action required.

* possibly related were: epistaxis, nasal pruritus, malaise.

^#^ probably related were: nasal discomfort, throat irritation.

## 4 DISCUSSION

In this clinical trial, a therapeutic equivalence of the novel low‐dose aqueous budesonide nasal spray “Budesolv 10” (200 µg/mL) and the marketed Rhinocort® Aqua 64 (1280 µg/mL) was demonstrated in allergic rhinitis patients after 8 days of treatment.

Despite the low dose of daily administered Budesolv 10 (40 µg/d) compared to 256 µg budesonide delivered by RA, both treatments led to a mean reduction of more than 2 TNSS points after 8 treatment days. Furthermore, Budesolv demonstrated non‐inferiority and thus can be considered equivalent to RA. Non‐inferiority was not only shown for TNSS as subjective parameter, but also for anterior nasal airflow measured by active rhinomanometry. Likewise, data obtained for nasal secretion weight supported the non‐inferiority evidence.

In general, these results are not surprising, as dose titrations with nasal sprays containing 64 µg or 256 µg budesonide per application (two puffs in each nostril resulting in 100 µL per nostril) hardly showed any short‐term differences (within the first 12 hours after application), suggesting that the dissolved part of the formulation is relevant for the response.^5^ In a comparison of 128 µg to 256 µg budesonide per day over four to six weeks, a slightly better response was observed for the higher dose; however, both concentrations resulted in a substantial or total control of the symptoms in 88.4% or 85.3% of treated patients, respectively. In the same study, fluticasone propionate was tested (200 µg per day), which showed a consistently lower responses than 256 µg/d Budesonide.^16^ This is of particular interest, as fluticasone has a higher affinity to the glucocorticoid receptor and a stronger activation capacity to induce corticosteroid‐dependent mRNA transcription than budesonide.^17^, ^18^ This higher activity, however, does not translate into higher activity in patients, most probably because fluticasone is even less bioavailable than budesonide. These data together suggest that the limiting factor for efficacy is solubility of the drug and the resulting bioavailability in the respective tissue on the one hand, as well as the availability of the respective receptor in the target tissue.

Budesolv 10 is an aqueous micellar solution of 200 µg/mL of budesonide. Preclinical and clinical data indicate that the treatment with this novel formulation resulted in a higher local availability compared to marketed products. Although Budesolv allows an increased local bioavailability, systemic exposure of budesonide cannot exceed the levels observed after treatment with RA. For RA, it has been shown that approximately 30% of the applied drug is found in the plasma.^18^, ^19^ As Budesolv only contains less than 16% of budesonide compared to RA, systemic levels as observed after treatment with RA cannot be reached. The bioavailability is too low to result in a measurable systemic concentration, particularly at the low dose applied. The portion that is swallowed is presumably metabolized as know from budesonide including a cytochrome C‐dependent inactivation in the liver.

In this study, it was furthermore shown that Budesolv 10 exerts a fast and clinically relevant onset of therapeutic efficacy after an initial single treatment.

As glucocorticoids are highly lipophilic substances, most current nasal spray formulations are presented as suspensions. Because only dissolved drugs can cross the membrane and become bioavailable, any solid undissolved compound may be removed from the nasal cavity by physiological mechanical clearing mechanisms.^4^ Consequently, the peak effectiveness of current glucocorticoid containing nasal sprays is achieved only after several days of treatment with multiple applications, which makes these medications a potent prophylactic and preventive treatment but an unsatisfying medication for acute treatment. Particularly in seasonal allergic rhinitis, a prophylactic treatment is difficult to achieve because it is not obvious when and to which extent the challenging antigen is present. In fact, the patient information leaflet of RA recommends a treatment period of at least 7 days to obtain maximal therapeutic efficiency.

In several clinical field and challenge chamber studies, immediate improvement of allergic rhinitis symptoms could be shown only with fixed combination products containing an antihistamine compound. Comparisons of intranasal steroid, intranasal antihistamine and a fixed combination of both revealed an onset of action within 90 minutes for the antihistamine containing preparations only, but not for the corticosteroid compound.^6^, ^20^ After a minimum of 8 days of treatment, a clear add‐on effect of the fixed combination compared to mono‐substances alone was evident indicating an effect size of 19% ^21^ and above ^6^, ^20^, ^22^ which is comparable to the results of our study.

However, the current study was designed to show non‐inferiority to RA‐ therefore, a treatment period of 8 days was chosen. Budesolv 10 was developed to provide the opportunity to treat acute symptoms of allergic rhinitis; this was investigated on day 1 of the study. A challenge chamber approach was chosen to capture and document the development of acute allergic rhinitis symptoms and the onset of action of the evaluated investigational products as precise as possible. A minimum qualification score was set as selection criterion at screening only to include solely patients with at least moderate clinical reactivity for this study. Under natural environmental conditions, patients experience some variability in their clinical symptoms as well, what needs to be considered during sample size calculation already. However, TNSS curves of repeated challenges in the VCC show a consistent reproducibility ^23^ being evident also in this study (data not shown).

Remarkably, an initial single treatment with 40 µg of soluble budesonide significantly reduced the summarized mean TNSS from 90 minutes onwards (see Figure 4A) throughout the entire ECC session. When the mean TNSS was compared before and after (ie at the end of observation period, 4:15 hours) treatment, a mean reduction of 1.24 (95% CI; −1.81‐−0.66) symptom score points was observed. Interestingly, this reduction was already 50 percent of the maximum achievable reduction in TNSS on day 8. Devillier et al used three different anchor‐based methods and two distribution‐based methods to estimate the minimally important difference in rhinoconjunctivitis total symptom score (RTSS) in children, adolescents and adults suffering from grass pollen–induced AR. They concluded that a change of at least 1 RTSS point can be considered as minimally important difference.^24^ Budesolv reached this clinically significant 1 point reduction in mean TNSS as early as 195 minutes (3:15 hours) after application. Furthermore, according to the FDA guidance for industry recommendation, our data showed an significant early onset of action of Budesolv 10 at 90 minutes after initial treatment.^25^ The fast onset of action of Budesolv 10 is supported by our preclinical data showing an immediate local budesonide bioavailability in porcine nasal mucosa when applied in form of Budesolv (Nakowitsch et al, manuscript in preparation) compared to RA. Non‐genomic effects of glucocorticoids take place fast, within minutes or even seconds, in the time excluding protein production de novo. These effects include regulation of membrane ion channels, regulation of T‐cell receptor (TCR) signalling, effect on G protein signalling and stimulation the release of Src kinase.^26^ Surprisingly, Budesolv 10 treatment had an immediate therapeutic effect on respiratory asthma‐like symptoms, such as wheeze, cough and dyspnoea, from 15 minutes onwards. Within 4 hours after initial treatment, a TRSS reduction of the same extent as achieved on day 8 was observed. Since AR and asthma frequently coexist in the same subjects, this represents a global health problem. While 10%‐40% of individuals with allergic rhinitis have asthma, over 80% of asthmatics suffer from allergic rhinitis. AR is considered an independent risk factor for asthma ^27^ with an adjusted odds ratio of 3.21.^3^ Some studies have shown that treatment for AR can reduce healthcare costs and lead to a better asthma control.^28^, ^29^ In the future, the challenge of AR care will be to optimize care pathways leading to a higher level of symptom control and prevent the progression towards asthma. Given the relationship between allergic rhinitis and asthma, it can be hypothesized that reducing inflammation in the upper airway with intranasal corticosteroid medications may have a positive effect on the onset of asthma. In fact, a systematic review of that subject found that intranasal corticosteroids improve some asthma‐specific outcome measures in patients suffering from both allergic rhinitis and asthma.^30^ After a treatment period of at least 4 weeks, the six pooled trials reported a significant improvement in respiratory symptom scores of 0.42 (95% CI; ‐0.53‐‐0.03). In our study, we found a reduction in total respiratory symptom score of 0.25 (95% CI; ‐0.45‐‐0.06) score points after an initial single treatment with Budesolv 10 when all participants were evaluated (reduction of 45% compared to placebo). If only participants showing any respiratory symptoms (ITT_resp,_ n = 57) were considered, the respiratory symptom score was reduced by 0.51 score points (reduction of 47% compared to placebo); if only volunteers that showed a TRSS of more than 1 score point were included for analysis (n = 38), the TNSS reduction was as high as 0.9 score points. Our hypothesis for the somewhat unexpected finding of a fast and strong suppression of respiratory symptoms by the application of Budesolv 10 is the increased availability of budesonide in the pharyngeal and upper airway mucosa of the respiratory tract, where reduction in oedema and irritation could have led to reduced subjective respiratory symptoms. This hypothesis was supported by the fact that total ocular symptom score was not affected by Budesolv 10 treatment on day 1. The interaction between upper and lower airways has been discussed repeatedly in the literature, and several potential mechanisms can be considered accordingly: a nasobronchial reflex was described several decades ago already showing that nasal provocation induces an immediate bronchial reaction as well.^31^, ^32^ A comparable interaction could be proven for effector cell markers when a nasal provocation induced upregulation of ICAM‐1, VCAM‐1 and E‐selectin also in the lower airways ^33^ or a segmental bronchoprovocation in non‐asthmatic allergic rhinitis patients led to mast cell degranulation and increase of basophils in the nasal mucosa, too.^34^

To conclude, Budesolv 10, the novel aqueous formulation of the corticosteroid budesonide, demonstrated non‐inferiority to RA in this pivotal phase III study. The data showed that solubilization of budesonide allows sparing of more than 84% of drug relative to the originator product RA, while still resulting in a non‐inferior clinical outcome with respect to TNSS on day 8, the primary end‐point. Consequently, the total exposure of patients to the drug was drastically reduced. The aqueous formulation is also free of preservatives such as benzalkonium chloride, paraben or phenyl‐ethyl‐alcohol, which reportedly have negative effects in the nasal cavity.^35^, ^36^ Moreover, treatment with Budesolv 10 led to a fast relief of allergic rhinitis symptoms, reported as TNSS. In addition, Budesolv 10 appears to be the first intranasal formulation of budesonide that reveals a significant effect on respiratory symptoms, exemplified by cough, wheezing and dyspnoea. Thus, Budesolv 10 can be a promising new option for the acute therapeutic treatment of seasonal and perennial allergic rhinitis with or without AR comorbidities like asthma and conjunctivitis.

## 5 CONFLICT OF INTEREST 

The authors declare the following financial interests/personal relationships which may be considered as potential competing interests: PZ, RZ, RS, PL and MS declare that they have no known competing financial interests or personal relationships that could have appeared to influence the work reported in this paper. NU, AG, EP and SN are employees of Marinomed.

## Supporting information

Supplementary Material
