# Supplementary material for: Fast effectiveness of a solubilized low‐dose budesonide nasal spray in allergic rhinitis
Source: Clin Exp Allergy. 2020 Jul 7;50(9):1065–77. doi: 10.1111/cea.13691 (PMC7540702; doi:10.1111/cea.13691)
Supplement: Supplementary file 1 [file CEA-50-1065-s001.docx]

Supplemental Material:

Table 2S

Adverse events grouped by system organ class, preferred term and severity; Safety set

SOC / PT	not related	unlikely	possible	probable	To
**Ear and labyrinth disorders**	**1**				
Middle ear inflammation	1				
**Gastrointestinal disorders**	**2**				
Abdominal pain upper	1				
Toothache	1				
**General disorders and administration site conditions**	**1**		**1**		
Malaise			1		
Pyrexia	1				
**Endocrine disorders**	**6**	**1**			
Allergy to animal	2				
Drug hypersensitivity		1			
Hypersensitivity	4				
**Infections and infestations**	**13**				
Bronchitis	1				
Nasopharyngitis	10				
Tonsillitis	2				
**Investigations**	**1**				
Gastric pH decreased	1				
**Musculoskeletal and connective tissue disorders**	**5**				
Back pain	4				
Pain in extremity	1				
**Nervous system disorders**	**9**				
Headache	8				
Migraine	1				
**Reproductive system and breast disorders**	**3**				
Dysmenorrhoea	3				
**Respiratory, thoracic and mediastinal disorders**	**8**	**2**	**4**	**3**	
Cough	3				
Dysphonia	1				
Epistaxis		1	3		
Nasal congestion	2				
Nasal discomfort				2	
Nasal pruritus			1		
Oropharyngeal pain	2				
Throat irritation		1		1	
**Skin and subcutaneous tissue disorders**		**1**			
Pruritus		1			
**Total**	**49**	**4**	**5**	**3**	
